# Changes in Invasive *Neisseria meningitidis* and *Haemophilus influenzae* Infections in France during the COVID-19 Pandemic

**DOI:** 10.3390/microorganisms10050907

**Published:** 2022-04-26

**Authors:** Ala-Eddine Deghmane, Muhamed-Kheir Taha

**Affiliations:** Invasive Bacterial Infections, Institut Pasteur, 75724 Paris, France; ala-eddine.deghmane@pasteur.fr

**Keywords:** invasive bacterial infections, epidemiology, COVID-19, *Neisseria meningitidis*, *Haemophilus influenzae*, vaccination

## Abstract

**Background**Since the appearance of COVID-19 in January 2020, invasive bacterial infections have decreased significantly worldwide. However, alterations in age and sex distributions, clinical forms, phenotypes, and genotypes of isolates have not been analyzed. Our goal is to present and discuss these data considering the current COVID-19 pandemic situation. **Methods:** The data of the national reference center for meningococci and *Haemophilus influenzae* in France were mined to examine the above aspects of invasive bacterial infection before (2018–2019) and after (2020–2021) the COVID-19 pandemic. Detailed epidemiological, clinical, and microbiological data were collected, and whole genome sequencing was carried out on meningococcal isolates (*n =* 1466). **Results:** In addition to the overall decline in the number of cases, various changes in age, sex, and phenotypes of isolates were also noted. As for *N. meningitidis*, more cases were observed in adults, as well as more invasive pneumopathies. Furthermore, fewer hyperinvasive meningococcal genotypes have circulated since COVID-19 emerged. The situation has been different for *H. influenzae*, as the number of invasive cases among adults decreased due to a reduction in non-typeable isolates. In contrast, cases due to serotypeable isolates, particularly serotypes a and b, increased in children <5 years-old. **Conclusions:** It is possible that measures implemented to stop COVID-19 may have reduced the circulation of *N. meningitidis* and *H. influenzae* isolates, but to a variable extent. This may be due to differences in circulation between these two species according to age groups. Vaccination schedules against these two species may have also influenced the evolution of these invasive bacterial infections since the emergence of the COVID-19 pandemic.

## 1. Introduction

The World Health Organization (WHO) officially declared the COVID-19 pandemic on week 11 of 2020. Thereafter, several countries implemented restriction measures, but with variable stringency, to reduce viral transmission, such as lockdown, social and physical distancing, an economic response, and health system organization. These measures also correlated with a sharp reduction in the incidence of several invasive bacterial infections (IBI). Data from 26 worldwide countries and territories showed that cumulative curves from January to 31 May 2020, revealed a drastic decrease in the number of isolates of invasive infection caused by bacteria such as *Haemophilus influenzae* (Hi), *Neisseria meningitidis* (Nm), and *Streptococcus pneumoniae* (Sp), but no change was observed for IBI cases provoked by group B streptococci. This reduction was observed regardless of the degree of stringency of the implemented measures [1]. In France, as in other countries, this reduction continued even after the end of the first lockdown between the 16 March and 15 May 2020 and concerned all meningococcal serogroups. However, invasive meningococcal disease (IMD) with respiratory presentations significantly increased during the period of the first lockdown (16 March–15 May 2020) compared to the same periods of 2018 and 2019 [2]. Although this change was observed in the elderly, no detailed data were reported on age distribution, in particular after almost two years of the pandemic and several waves of COVID-19 and several sets of containment measures. The reduction in cases of IBI (Hi, Nm, and Sp) was suggested to be due to the lower circulation of these bacterial agents that are mainly transmitted by respiratory pathways, upon the implementation of containment policies [1]. Moreover, this lower circulation was associated during this period with lower vaccine uptake including childhood vaccines against Hi, Nm, and Sp [3,4]. The combination of these factors was suggested to have provoked “an immunity gap” in the population that was predicted to be responsible for a “rebound” in the cases of IBI upon the release of containment measures [5]. 

As the containment measures have started to become less stringent in several countries, we aimed in this work to describe the evolution of IBI due to Hi and Nm according to age and serotypes/serogroups in France during the last five years (2017–2021).

## 2. Materials and Methods 

### 2.1. Study Design and Identification of Meningococcal and Hi Isolates

IMD and invasive Hi disease (IHiD) are defined on the basis of detection of Nm and Hi in normally sterile sites. Epidemiological surveillance of IMD and IHiD in France relies in part on sending clinical bacterial isolates and primary samples to the national reference center for meningococci and *Haemophilus influenzae* (NRCMHi) for identification, confirmation, and full typing. All data and materials were collected as part of the mission of surveillance conducted by the NRCMHi, and the procedure of collecting samples and information was submitted and approved by the CNIL N°1475242/2011 (*Commission Nationale de l’Informatique et des Libertés*). *N. meningitidis* and *H. influenzae* identification, grouping/typing, and genotyping, including whole genome sequencing, were performed as previously described [6,7]. The genotypes of meningococci, including the sequence types and the clonal complexes, were extracted from whole genome sequencing data using the available tools on www.pubmlst.org accessed on 31 January 2022 [8]. The genomic data (FASTA files) can be retrieved from the PUBMLST.org site by filtering on country (France) and period (years 2017–2021). Data on the number of COVID-19 cases were from the WHO (https//covid.who.int/data; accessed on 18 April 2022). 

### 2.2. Statistical Analysis

Numbers and percentages of total isolates by serogroups (Nm) and serotypes (Hi) were presented. Analyses by age intervals were also calculated for the distribution of both bacterial species. The same age groups were used for both bacterial species (<1 year, 1–4 years, 5–14 years, 15–24 years, 24–44 years, 46–64 years, and ≥65 years). Chi-square or unpaired *t*-test were used to test the significance of differences in categorical or continuous variables when appropriate. Statistical analyses were performed using Prism GraphPad Software (Version 9.1.2), and a two-tailed cut-off of a *p*-value of 0.05 was considered statistically significant whenever applicable. 

## 3. Results

### 3.1. Evolution of IMD Cases during the Period 2017–2021

**Figure 1:**
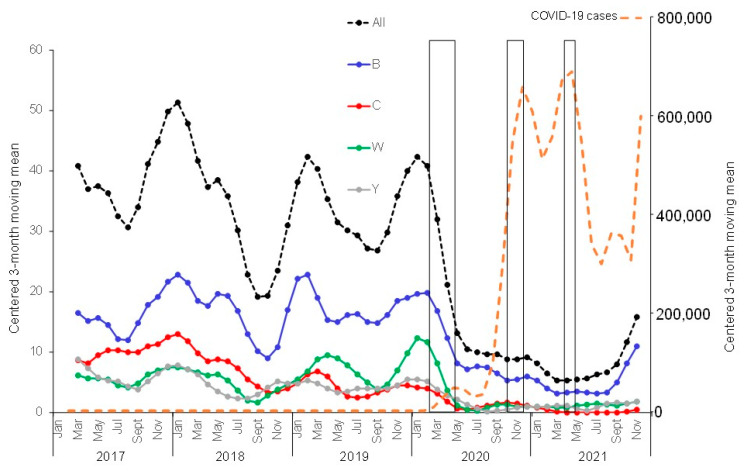
Evolution of IMD cases by serogroup. Data are expressed as the centered 3-month moving means of the number of cases per serogroup. Serogroups are indicated in different colors. Note that the first lockdown was implemented in France on 15 March 2020. COVID-19 cases were also expressed using 3-month moving means (right axis). The three lockdown periods are indicated by boxes (15 March–15 May 2020; 30 October–15 December 2020; 3 April–3 May 2021).

**Figure 2:**
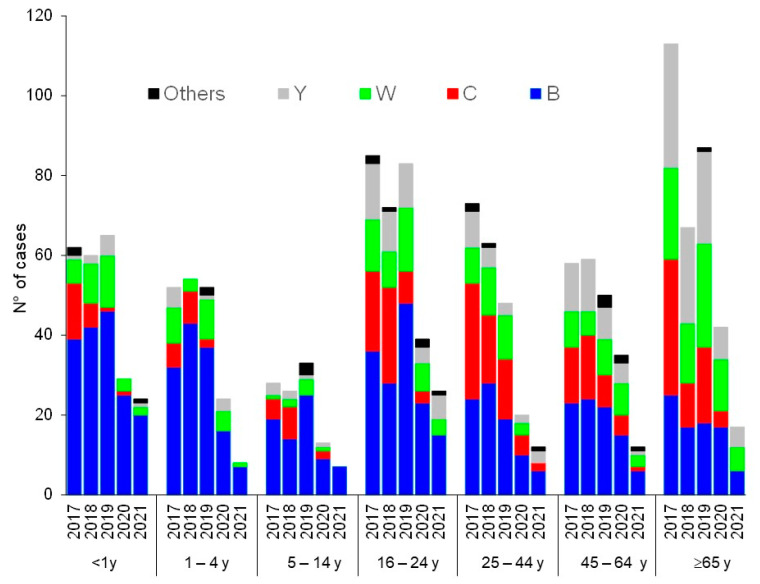
Evolution of number of cases of IMD over the period 2017–2021 per age group and per serogroup.

**Figure 3:**
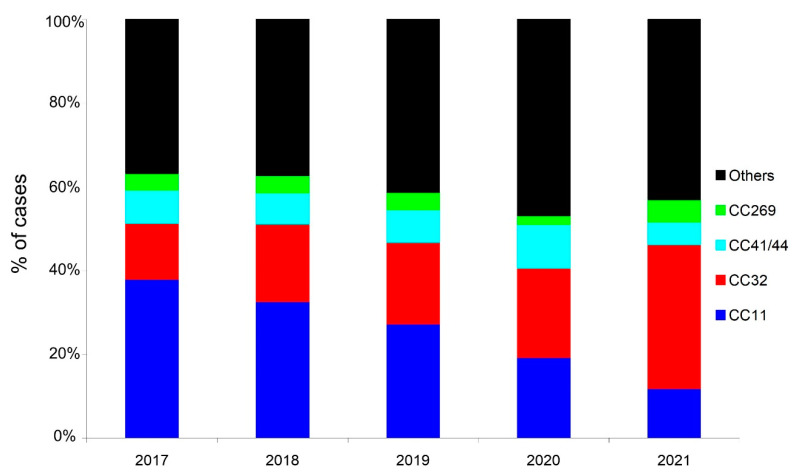
Evolution of cases of IMD in percentage per clonal complexes (indicated on the right).

The NRCMHi received and characterized a total of 1595 IMD cases from 2017 to 2021 (474, 397, 416, 202, and 106 cases, respectively). There were 791 serogroup B cases (49.6%), 286 serogroup C cases (17.9%), 271 serogroup W cases (17%), 221 serogroup Y cases (13.9%), and 26 cases of other groups and non-groupable cases (1.6%). The yearly distribution of the serogroups over the studied 5-year period showed decreasing numbers of cases since the declaration of the COVID-19 pandemic in comparison to the period prior to this pandemic, although the decrease seemed to be less prominent in 2021 compared to 2020. Indeed, when the centered three-month moving means (that clearly show the seasonal variation of IMD) were compared for the four major serogroups, the sharp decrease was observed in spring 2020 for serogroups B, W, and Y, but this seemed to reverse during the second half of 2021, where cases started to increase again (Figure 1). Similar trends were also observed when the centered twelve-month moving mean was used for the four major serogroups (Figure S1). For serogroup C, the decrease started earlier, in the spring of 2018, and continued into the years 2020 and 2021. Age and serogroup distributions of cases indicate that serogroup B remains the most frequent serogroup in all age groups (except for 65 years and older). The deceleration observed in the second half of 2021 (Figure 1) was mainly observed among infants <1 year (Figure 2). We finally analyzed the genotypes of the circulating isolates during the studied period. Typing data were available for 1466 cases of the 1595 cases of IMD (92%). The hyperinvasive clonal complexes (CC11, CC32, CC41/44, and CC269) were scored. The other clonal complexes and the non-assigned isolates were scored in one group (Figure 3). The most prominent trend was the decreasing proportion of isolates belonging to the hyperinvasive clonal complexes that was mainly due to the decrease in isolates of CC11. CC32 and others showed, conversely, an increasing trend (Figure 3).

### 3.2. Evolution of Invasive Hi Disease Cases during the Period 2017–2021

**Figure 4:**
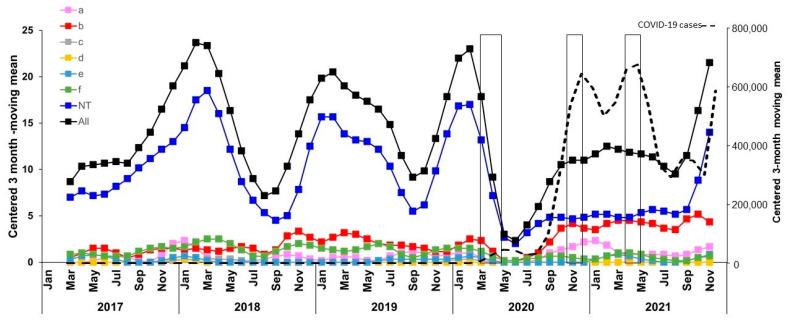
Evolution of IHiD cases by serotype. Data are expressed as the centered 3-month moving means of the number of cases per serotype. Serotypes are indicated in different colors. COVID-19 cases were also expressed using 3-month moving means (right axis). The three lockdown periods are indicated by boxes (15 March–15 May 2020; 30 October–15 December 2020; 3 April–3 May 2021).

During the period 2017–2021, a total number of 808 IHiD cases were received and characterized at the NRCMHi (137, 177, 189, 132, and 173 cases in 2017, 2018, 2019, 2020, and 2021, respectively). Overall, non-capsulated (non-typable, NTHi) isolates accounted for 550 cases (68.1% of all isolates for the whole 2017–2021 period). The annual numbers of NTHi were 99, 128, 138, 87, and 98 in 2017, 2018, 2019, 2020, and 2021, respectively. The centered 3-month moving mean of IHiD cases suggests that the seasonal pattern of cases that is usually observed in the winter months (December–March) was lost in 2020. A sharp decrease in cases was observed from March 2020 for all Hi isolates (both typeable and non-typeable isolates) (Figure 4). A similar observation was made using centered twelve-month moving mean for the Hi invasive isolates (Figure S2).

**Figure 5:**
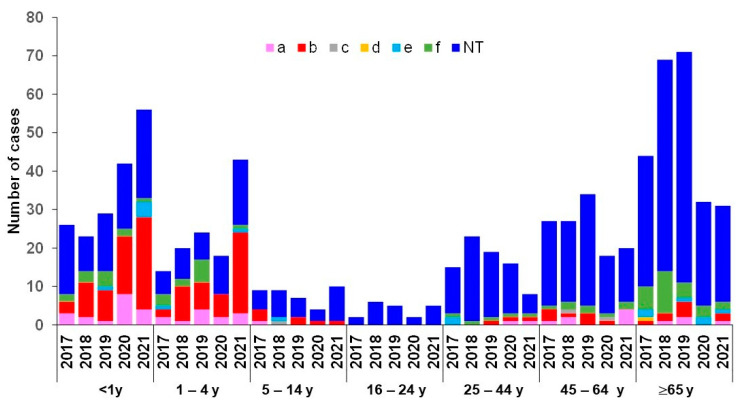
Evolution of number of cases of IHiD over the period 2017–2021 per age group and per serotype.

The decline in invasive disease cases observed in 2020 resulted mainly from NTHi cases. The decrease continued in 2021 for NTHi isolates, albeit to a lesser extent. NTHi decreased in proportion from 73% (365/503) in 2017–2019 to 66% (87/132) in 2020 and to 57% (98/173; *p* = 0.0001) in 2021 (Figure 4). NTHi isolates were the most common cause of disease in all age groups, particularly among the 65 year-olds, where they accounted for 37% of cases (201/550, *p* < 0.0001). The decrease in NTHi cases observed since 2020 was more pronounced in adults 45 years and over (both 45–64 and ≥65 year-olds). Indeed, NTHi isolates were detected at an average of 74 cases per year among subjects ≥45 year-olds during the period 2017–2019. This number decreased by 43% in 2020 (42 cases) and then by 7% (39 cases) in 2021 (Figure 5).

However, serotypeable invasive isolates, after a short period of decrease between March and July 2020, increased (in particular, isolates of serotypes a and b) as shown by the centered 3-month moving mean of IHiD cases (Figure 4).

In contrast to adults, the proportion of all invasive cases among children <5 years old increased from 27% (136/503) in 2017–2019 to 45% (60/132) in 2020 and 57% (99/173) in 2021, despite the COVID-19 containment measures (Figure 5). In particular, of the 131 Hib cases, 79% (104/131) occurred among children <5 years old, of which 57% (59/104) were among infants <1 year. During the period 2017–2019, Hib cases represented 28% (38/136) of all invasive disease among children <5 years old. This proportion increased to 35% (21/60) in 2020 and reached 45.5% (45/99) in 2021. As for serotype b, Hia occurred more frequently (30/44, 68%) among children <5 years old. During the period 2017–2019, Hia caused 10% (13/136) of all cases in children <5 years old. This proportion increased to 17% (10/60) in 2020 but declined thereafter to 7% (7/99) in 2021 (Figure 5).

## 4. Discussion

The COVID-19 pandemic has profoundly changed the epidemiology of invasive bacterial infections, and especially those provoked by respiratory pathogens [1]. Here, we provide 5-year data (2017–2021) supporting trends in invasive meningococcal and *H. influenzae* diseases. This may be the result of a combination of several factors [1]. Strict social restrictions intended to reduce the burden of SARS-CoV-2 infections were implemented, including national and local lockdowns, universal outdoor masking, and social distancing [1]. *H. influenzae* and *N. meningitidis* are most frequently airborne communicable pathogens. Our data suggest that a reduction in the circulation of and the exposure of individuals to these bacterial agents has been observed since the implementation of the first lockdown in France in March 2020. Our data are in line with the drastic decrease in notifications for a wide range of infectious diseases with a respiratory transmission route, including flu virus and RSV, observed during a similar time period in France [9]. The reduction in viral infections such as flu may potentiate the reduction in IBI due to the spatiotemporal association between flu and IMD that has been consistently reported [10,11]. A model of secondary bacterial infection in flu-infected mice was also developed [12]. The relative importance of these two factors (reduction in the circulation of bacterial agents and the reduction in viral infections) may vary according to the bacterial agents. The reduction in *Streptococcus pneumoniae*-associated diseases was suggested to be more linked to the reduction in several respiratory viruses [13]. According to our data, reduced circulation may have a greater effect as cases of IMD decreased mainly due to highly transmissible isolates, such as CC11. 

The reduction in IBI cases also occurred in the context of a decreasing trend of vaccine uptake during the pandemic that was suggested to be due to several factors (uncertainty of parents about whether vaccinations were taking place, difficulties in having appointments in medical rooms as well as school closures) [3]. Additionally, the simplification of vaccination strategies in several countries such as France reduced the number of primary doses of vaccines against respiratory pathogens (such as Hi of serotype b, Hib, and *Bordetella pertussis*) by switching from the 3 + 1 to 2 + 1 schedule [14]. These changes in vaccination strategies were suggested to be associated with an increase in the number of *B. pertussis* and Hib cases [15,16]. The combined effect of all these factors (less bacterial circulation, lower vaccine uptake, and lower number of doses) may have provoked an immunity gap/immunity debt [5]. The reduced bacterial circulation also led to a decline in IBI cases. However, easing restrictions may lead to a rebound in IBI cases in sub-optimally immunized populations. Our data suggest that after the decreasing phase of IHiD cases in 2020, cases started to increase in 2021 among children less than 5 years who presented with the greatest increase in IHiD among all groups examined in this study, in particular, IHiD associated with Hib and, to a lesser extent, with Hia. 

By easing social restrictions since the second half of 2020, individuals, in particular children, returned to day care and schools. This was associated with a rebound of the disease. Recent data from England and Wales reported an increase in the number of IMD cases among adolescents and young adults, but not in children <5 years old who are targeted by vaccination against meningococci of serogroup B, unlike adolescents and young adults. Our data suggest that for serogroups B, W, and Y, the IMD cases started to increase again during the second half of 2021, in particular, among those <1 year old [17,18]. No general vaccination was implemented in France against serogroups W and Y. A recent recommendation (June 2021) against serogroup B was announced but is still to be implemented [19]. Interestingly, our data showed the number of IMD cases due to serogroup C started to decline in 2018 upon the implementation of mandatory vaccination among children <2 years of age. These cases continued to decrease during the COVID-19 pandemic. 

## 5. Conclusions

IBI, and in particular IMD, is typically described as changing in a stochastic manner [20]. However, the epidemiology of IBI caused by respiratory pathogens, such as Nm and Hi, has shown a drastic decline since the emergence of the COVID-19 pandemic. IBI surveillance is essential for readapting vaccination strategies and monitoring any resurgences of these diseases.

## Data Availability

The genomic data (FASTA files) for *N. meningitidis* can be retrieved from the PUBMLST.org site by filtering on country (France) and period (years 2017–2021).

## Supplementary Materials

The following supporting information can be downloaded at: https://www.mdpi.com/article/10.3390/microorganisms10050907/s1, Figure S1: Evolution of IMD cases by serogroup. Data are expressed as the centered 12-month moving means of the number of cases per serogroup. Serogroups are indicated in different colors. Note that the first lockdown was implemented in France on 15 March 2020, Figure S2: Evolution of IHiD cases by serotype. Data are expressed as the centered 12-month moving means of the number of cases per serotype. Serotypes are indicated in different colors.
