# Supplementary material for: Changes in Invasive Neisseria meningitidis and Haemophilus influenzae Infections in France during the COVID-19 Pandemic
Source: Microorganisms. 2022 Apr 26;10(5):907. doi: 10.3390/microorganisms10050907 (PMC9147110; doi:10.3390/microorganisms10050907)
Supplement: Supplementary file 1 [file microorganisms-10-00907-s001.zip › microorganisms-1669855-supplementary.pdf]

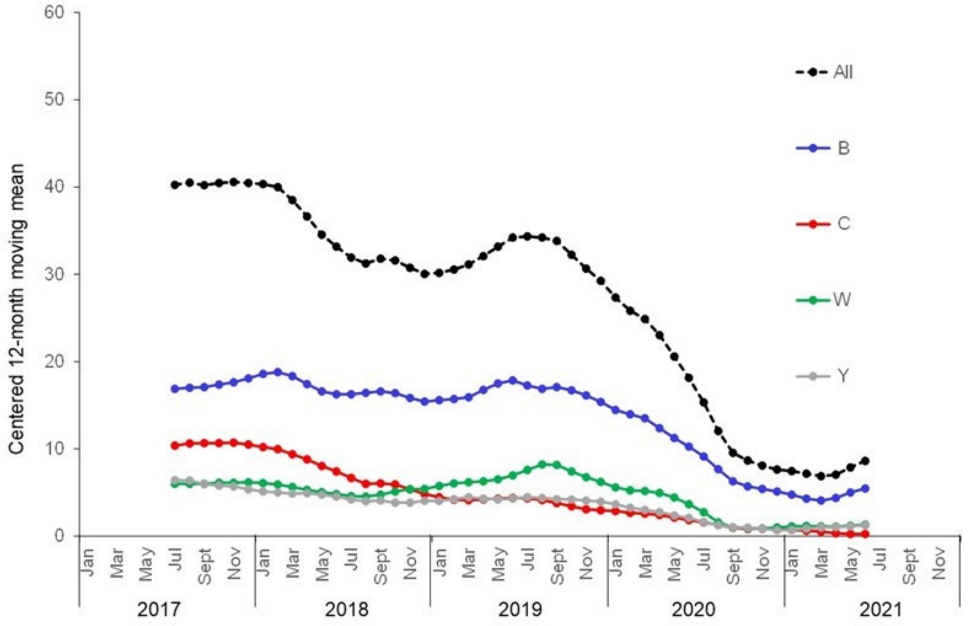

Figure S1: Evolution of IMD cases by serogroup. Data are expressed as the centered 12-month moving means of the number of cases per serogroup. Serogroups are indicated in different colors. Note that the first lockdown was implemented in France on 15 March 2020.

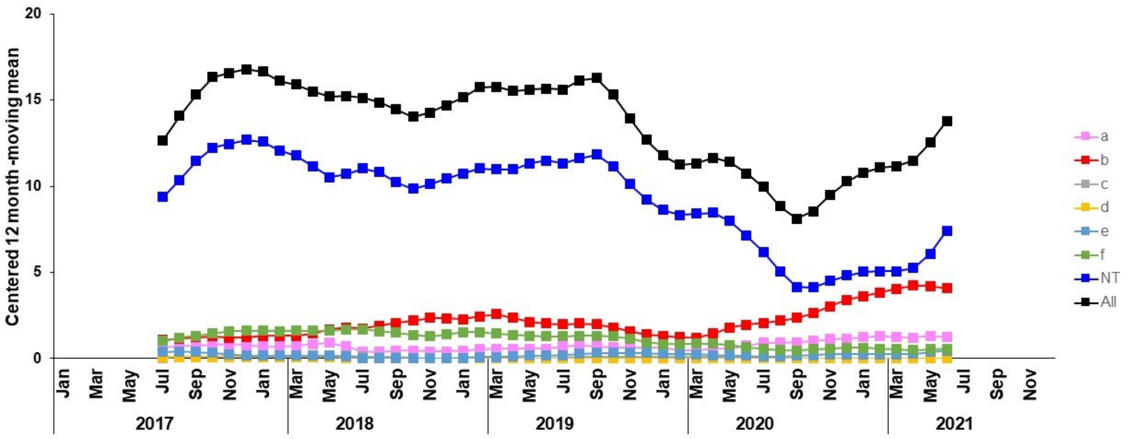

Figure S2: Evolution of IHiD cases by serotype. Data are expressed as the centered 12-month moving means of the number of cases per serotype. Serotypes are indicated in different colors.
